# Covid-19 vaccination coverage and associated factors among older hypertensive patients in Hangzhou, China

**DOI:** 10.1093/inthealth/ihae019

**Published:** 2024-02-15

**Authors:** Shijun Liu, Caixia Jiang, Yan Liu, Xin Qiu, Jun Luo, Jing Wang, Yuyang Xu

**Affiliations:** Department of Non-communicable and Chronic Diseases, Hangzhou Center for Disease Control and prevention, Mingshi Road No. 568, Hangzhou, Zhejiang 310021, China; Department of Non-communicable and Chronic Diseases, Hangzhou Center for Disease Control and prevention, Mingshi Road No. 568, Hangzhou, Zhejiang 310021, China; Department of Non-communicable and Chronic Diseases, Hangzhou Center for Disease Control and prevention, Mingshi Road No. 568, Hangzhou, Zhejiang 310021, China; Department of Non-communicable and Chronic Diseases, Hangzhou Center for Disease Control and prevention, Mingshi Road No. 568, Hangzhou, Zhejiang 310021, China; Department of Non-communicable and Chronic Diseases, Hangzhou Center for Disease Control and prevention, Mingshi Road No. 568, Hangzhou, Zhejiang 310021, China; Department of Expanded Program on Immunization, Hangzhou Center for Disease Control and Prevention, Mingshi Road No. 568, Hangzhou, Zhejiang 310021, China; Department of Expanded Program on Immunization, Hangzhou Center for Disease Control and Prevention, Mingshi Road No. 568, Hangzhou, Zhejiang 310021, China

**Keywords:** COVID-19, COVID-19 vaccination, electronic health record, hypertensive patient, SARS-CoV-2 vaccine

## Abstract

**Background:**

Vaccination could provide effective protection against coronavirus disease 2019 (COVID-19). This study aims to describe the COVID-19 vaccination coverage and influential factors in Chinese older hypertensive patients.

**Methods:**

Using a cross-sectional design, participants were randomly selected from the electronic health records system during the pandemic era in Hangzhou, China. Logistic regression models were employed to compute the OR and 95% CI in order to assess the relationships between variables and the extent of COVID-19 vaccination coverage.

**Results:**

As of 3 August 2022, among a sample of 77 970 individuals, 75.11% had completed the full COVID-19 vaccination, while 57.66% had received a booster dose. Disparities in coverage were observed across genders, regions and age groups. Unhealthy lifestyles, cardiovascular disease, cancer, uncontrolled blood pressure, abnormal fasting plasma glucose, dyslipidemia and renal dysfunction were risk factors for COVID-19 vaccination coverage. The coverage rates continuously declined along with the number of risk factors. The ORs for full and booster vaccination in subjects with ≥4 risk factors were 2.55 (2.12∼3.07) and 2.60 (2.16∼3.13), compared to individuals without risk factors.

**Conclusion:**

The COVID-19 vaccination program for older hypertensive patients must be strengthened further. Emphasis should be placed on patients who reside in urban areas, have comorbidities or multiple risk factors.

## Introduction

The coronavirus disease 2019 (COVID-19) vaccination can provide effective protection against infection and death due to Severe Acute Respiratory Syndrome Coronavirus 2 (SARS-CoV-2).^1,2^ A study based on mathematical modeling estimated that, in the first year of COVID-19 vaccination, by 2021 approximately 31.4 million deaths related to the disease were prevented across 185 countries and territories.^3^ As early as 29 March 2021, the National Health Commission of the People's Republic of China issued a COVID-19 vaccination program for the public.^4^ Full vaccination was initially conducted in mainland China for adults aged 18 y and above from March 2021. A booster vaccination campaign was subsequently initiated among these populations from October 2021 onward. As at 10 August 2022, 90.4% of the population aged 60 y and older had received at least one dose of COVID-19 vaccine, with 85.6% and 67.8% having completed full and booster vaccinations, respectively.^5^

Studies have demonstrated a bidirectional relationship between COVID-19 and various comorbid conditions.^6,7^ Diseases such as hypertension diabetes, cardiovascular disease (CVD) and chronic kidney disease (CKD) have been identified as some of the most common risk factors for severe COVID-19 throughout the pandemic, and are also considered potential risk factors for long COVID in the post-COVID-19 era.^6,7^ A retrospective cohort study conducted during the initial COVID-19 pandemic in Wuhan, China found that hypertension was the most common comorbidity among diagnosed patients, and older age was associated with death in patients with COVID-19.^8^ Both older adults and hypertensive patients were vulnerable to COVID-19 infection and tended to suffer severe consequences and death, and they were prioritized groups in the COVID-19 vaccination campaign.^4,9,10^ The status of hypertension in China showed that 23.2% of the adult population (≥18 y) had hypertension, with low awareness, low treatment and poor control of hypertension, according to the China Hypertension Survey.^11^ Previous studies have indicated that adherence to public health advice and willingness for a COVID-19 vaccine were associated with the vaccination status.^12,13^ Based on the aforementioned situation, the Chinese government has consistently emphasized and broadly proposed that older adults and hypertensive patients receive the COVID-19 vaccination. However, current surveys on COVID-19 vaccination rates among older hypertensive patients in China are limited. This study utilized data from electronic health record (EHR) systems and electronic immunization systems, to assess the COVID-19 vaccination coverage in Chinese older hypertensive patients who were being managed by general practitioners (GPs) at community health centers (CHCs) during the pandemic era, as well as analyze factors influencing vaccination rates in this population.

## Materials and Methods

### Study design

This study was a cross-sectional survey. Participants were confirmed hypertensive patients who had participated in the Essential Public Health Service (EPHS) health management program in Hangzhou City, Zhejiang Province, China. The EPHS program in China is a national financial support project, proposed and approved by the National Health Commission, that aims to reverse the major health burden of priority populations such as hypertension or type 2 diabetes (T2DM) patients.^14^

### Sampling

On 29 July 2022, there were approximately 0.86 million hypertensive patients registered at 198 CHCs in Hangzhou city, according to the EHR system. A multi-stage stratified random sampling process was conducted using an automated procedure defined within the EHR system. First, hypertensive patients with T2DM were excluded from the EHR system, given that T2DM is a critical comorbidity associated with COVID-19.^6,15^ Second, based on our experience, the CHC and age group are two variables that have a significant impact on health management level. Therefore, patient categorization was conducted based on CHCs and followed by age group stratification with a 10-year interval per group. Finally, a random sampling method was utilized to select 20% of individuals aged 60 y and older from each CHC based on their age distribution. Subsequently, a total of 77 970 subjects with required variables were included, and 52 648 (67.18%) of these had a physical examination.

### Data source

An electronic immunization system was used to collect daily updates on COVID-19 vaccination information, primarily encompassing vaccination dose, vaccinated time and type of vaccine. The EHR system collected information including general characteristics, body measurements, lifestyle factors, laboratory testing, medication adherence, comorbidities. This analysis dataset was generated through a digital platform that combines EHR and immunization information. Vaccination information was updated until 3 August 2022.

### COVID-19 Vaccination

The varieties of COVID-19 vaccines in this study included inactivated virus vaccine (98.30%), recombinant protein vaccine (1.66%) and viral vector-based vaccine (0.04%). Full vaccination was defined as receiving two doses of inactivated virus vaccine, three doses of recombinant protein vaccine or one dose of viral vector-based vaccine. Booster vaccination was defined as receiving three doses of inactivated virus vaccine or two doses of viral vector-based vaccine. The administration of one dose of viral vector-based vaccine followed by two doses of inactivated virus vaccine is also defined as a booster vaccination. Receiving COVID-19 vaccination without the full course of doses is deemed incomplete vaccination. During the analysis of the booster vaccination, 811 subjects were excluded as official guidelines for the recombinant protein vaccine's booster program had not been issued prior to 3 August 2022.

### Variables and criterion

The diagnostic criteria for hypertension was SBP ≥ 140 or DBP ≥ 90 mmHg (1 mmHg = 0.133 kpa) or taking antihypertensive drugs within two weeks.^16^ The BP controlled thresholds for COVID-19 vaccination in hypertensive patients are as follows: For patients aged 60–65 y, the threshold is SBP < 140 mmHg and DBP < 90 mmHg. For patients aged 65 y and over, the threshold is SBP < 150 mmHg and DBP < 90 mmHg. Definitions of cardiovascular factors were conducted according to Chinese Guidelines for Prevention and Treatment of Hypertension.^16^ Smoking status is a dichotomous variable, and one cigarette per day at least during the past six months was considered current smoking. Weekly periods were used to define the frequency of alcohol drinking and physical exercise. Five days a week at least was defined as the ‘daily’ frequency. Current smoking, daily alcohol drinking or nondaily physical exercise were grouped as unhealthy lifestyles. Adherence to prescribed antihypertensive medications over the past six months was considered as regular medication adherence. The body mass index (BMI) was calculated as weight/height^2^ (kg/m^2^). BMI ≥ 28.0 kg/m^2^ and (or) waist circumference (WC) ≥90 cm (male), ≥85 cm (female) were defined as obesity. Dyslipidemia is a condition with total cholesterol (TC) ≥6.2 mmol/l, low density lipoprotein cholesterol (LDL-C) ≥4.1 mmol/l or high density lipoprotein cholesterol (HDL-C) <1.0 mmol/l. Fasting plasma glucose (FPG) ≥7.0 mmol/l was defined as abnormal FPG. Serum creatinine (Scr)  ≥ 133 μmol/l (male), ≥124 μmol/l (female) was defined as renal dysfunction. The regional distribution of CHCs was divided into urban, suburban and rural groups. Uncontrolled BP, unhealthy lifestyles, irregular medication adherence, obesity, dyslipidemia, abnormal FPG, renal dysfunction, CVD and cancer were considered risk factors affecting COVID-19 vaccination. As a result, subjects with or without a physical examination were divided into several groups according to the number of risk factors.

### Statistical analysis

Variables were introduced as overall subjects as well as those who underwent physical examination, and summarized using means with standard deviation for continuous data, percentages and proportions for categorized data. Due to the presence of overlap, the comparison between these groups was not conducted. The COVID-19 vaccination coverage (full and booster) was computed for the groups mentioned above, and presented in a monthly trend from April 2021 to August 2022. The coverage rates were further classified based on gender, region and age groups. Chi-squared test was used to compare the rates between groups. Logistic regression models were employed to calculate the OR and 95% CI for the associations between variables and the COVID-19 vaccination. The dependent variable was the status of full and booster vaccination (0 = complete, 1 = unvaccinated or incomplete). Separate models were fitted for the overall subjects as well as those who underwent a physical examination. For the overall subjects, independent variables included age, gender and urban–rural disparity, obesity, lifestyle, medication adherence and comorbidities. For the subjects who had a physical examination, additional independent variables included BP measurement, FPG, lipedema and Scr. Multivariable analysis was conducted by including all independent variables. Furthermore, the associations between the number of risk factors and COVID-19 vaccination were calculated while controlling for age, gender and urban–rural disparities. All statistical analyses were performed using R version 4.1.1 (The R Foundation for Statistical Computing, Vienna, Austria; https://www.R-project.org/). All p values were two-sided and statistical significance was defined as p<0.05.

## Results

### General characteristics

A total of 77 970 subjects with an average age of 72.74±6.96 y, of whom 46.83% were men and 53.17% were women. Nearly half of them were in the age groups ranging from 65 to 79 years old. The regional constitution distribution was 32.73% from urban areas, 52.50% from suburban areas and 14.77% from rural areas. The proportions of obesity, current smoking, daily alcohol drinking, and non-daily physical exercise were 40.43%, 16.25%, 12.79%, and 73.26%, respectively. In total 79.41% and 7.51% of subjects had unhealthy lifestyles and irregular medication adherence, with 19.18% and 5.97% combining this with CVD and cancer. For subjects who underwent physical examinations, additional measurements were calculated, including 37.33% with uncontrolled BP, 12.71% with abnormal FPG, 22.92% with dyslipidemia, and 3.49% with renal dysfunction (Table 1).

**Table 1.** General characteristics of subjects

	Groups	Overall subjects	Subjects with physical examination in 2021
N		77 970	52 648
Gender (%)	male	46.83	46.62
	female	53.17	53.38
Age (years)		72.74±6.96	72.82±7.81
Age groups (%)	60y∼	16.55	11.50
	65y∼	25.44	28.50
	70y∼	23.24	26.58
	75y∼	15.47	17.13
	80y∼	10.18	9.80
	85y∼	6.90	5.39
	90y∼	2.22	1.10
Region (%)	urban	32.73	25.55
	suburban	52.50	58.59
	rural	14.77	15.85
BMI (kg/m^2^)		24.32±3.28	24.41±3.25
WC (cm)		84.85±8.88	85.11±8.91
Obesity (%)	no	59.57	58.05
	yes	40.43	41.95
Current smoking (%)	no	83.75	84.20
	yes	16.25	15.80
Daily alcohol drinking (%)	no	87.21	88.02
	yes	12.79	11.98
Daily physical exercise (%)	yes	26.74	25.26
	no	73.26	74.74
Unhealthy lifestyle (%)	no	20.59	19.81
	yes	79.41	80.19
Medication adherence (%)	regular	92.49	92.24
	irregular	7.51	7.76
With CVD (%)	no	80.82	82.27
	yes	19.18	17.73
With cancer (%)	no	94.21	94.72
	yes	5.79	5.28
Uncontrolled BP (%)	no	—	62.67
	yes		37.33
Abnormal FPG (%)	no	—	87.29
	yes		12.71
Dyslipidemia (%)	no	—	77.08
	yes		22.92
Renal dysfunction (%)	no	—	96.51
	yes		3.49

### The coverage rates of COVID-19 vaccination

**Figure 1.**
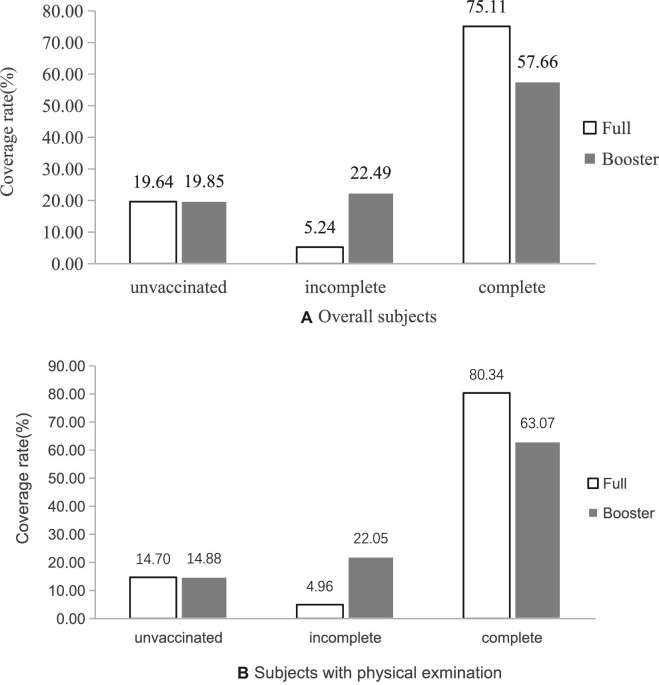
Coverage rates of full and booster COVID-19 vaccination.

**Figure 2.**
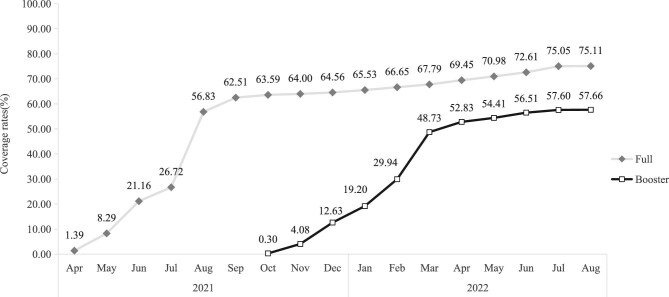
Temporal trend of COVID-19 vaccination coverage.

**Figure 3.**
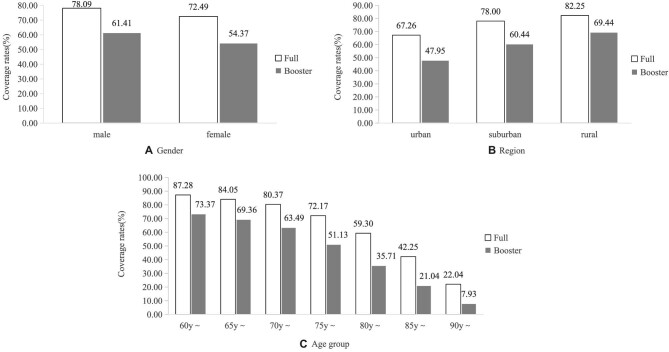
COVID-19 vaccination rates by gender, region and age groups.

The full and booster vaccination rates of COVID-19 were 75.11% and 57.66% for overall subjects, and 80.34% and 63.07% for subjects who had a physical examination (Figure 1). The temporal trend in coverage rates showed that the full vaccination rate increased from 1.39% in April 2021 to 62.51% in September 2021, then moved into a bottleneck period. The booster vaccination rate similarly increased from 0.30% in October 2021 to 52.83% in April 2022 (Figure 2). Higher coverage rates have been observed among males and in rural populations. Men received 78.09% of the full vaccination (χ^2^=325.73, p<0.001) and 64.41% of the booster vaccination (χ^2^=389.37, p<0.001). Rural subjects received 82.25% of the full vaccination (χ^2^=1338.90, p<0.001) and 69.44% of the booster vaccination (χ^2^=1746.39, p<0.001). Regarding age distribution, the 60 y∼ group exhibited the coverage highest rates of full vaccination (87.28%) and booster vaccination (73.37%), followed by a significant decrease in coverage among older age groups (χ^2^=8969.75, p<0.001; χ^2^=9095.29, p<0.001). For groups aged 85 y and older, the full rate was less than 50% and the booster rate declined to about 20% (Figure 3).

### Factors associated with coverage rates of COVID-19 vaccination

The logistic analysis was performed separately for overall subjects and those who had a physical examination in 2021. For overall subjects, the ORs (95% CI) of unhealthy lifestyles were 1.21 (1.16∼1.27) for full vaccination and 1.31 (1.26∼1.37) for booster vaccination compared to those with healthy lifestyles. Compared to those without comorbidities, subjects with CVD had ORs (95% CI) of 1.45 (0.139∼1.51) and 1.36 (1.31∼1.41), subjects with cancer had ORs (95% CI) of 2.71 (2.54∼2.90) and 2.23 (2.09∼2.39). In contrast, factors like obesity and medication adherence did not consistently associate with the full and booster COVID-19 vaccinations (Table 2). BP measurements and laboratory tests were added to the logistic analysis of the physical examination population. The uncontrolled BP with OR (95% CI) of 1.07 (1.03∼1.11) reduced the individual's acceptance of the COVID-19 booster vaccine. Abnormal FPG with OR (95% CI) of 1.28 (1.20∼1.37) and 1.28 (1.21∼1.36), dyslipidemia with OR (95% CI) of 1.08 (1.03∼1.14) and 1.05 (1.00∼1.10), and renal dysfunction with OR (95% CI) of 1.91 (1.72∼2.13) and 1.80 (1.62∼1.99) were risk factors for both full and booster vaccination (Table 2).

**Table 2.** Analysis of variables associated with COVID-19 vaccination coverage

		Coverage rate (%)						OR (95% CI)	
	Groups	Full	χ^2^	p Value	Booster	χ^2^	p Value	Full	Booster
Overall subjects (N=77 970)									
Obesity	no	74.59	17.05	<0.001	57.51	1.00	0.318	1	1
	yes	75.89			57.88			0.95 (0.91∼0.98)	0.99 (0.89∼1.03)
Unhealthy lifestyle	no	75.12	0.00	0.972	58.61	7.41	0.006	1	1
	yes	75.11			57.41			1.21 (1.16∼1.27)	1.31 (1.26∼1.37)
Medication adherence	regular	75.10	0.06	0.809	57.41	24.75	<0.001	1	1
	irregular	75.24			60.77			1.08 (1.01∼1.15)	0.94 (0.89∼1.03)
With CVD	no	77.67	1069.61	<0.001	84.73	946.98	<0.001	1	1
	yes	64.35			45.89			1.45 (1.39∼1.51)	1.36 (1.30∼1.41)
With cancer	no	76.28	931.53	<0.001	58.75	610.60	<0.001	1	1
	yes	56.05			39.97			2.72 (2.55∼2.91)	2.23 (2.09∼2.39)
Subjects with physical examination (N=52 648)									
Obesity	no	80.31	0.06	0.806	63.56	7.37	0.007	1	1
	yes	80.39			62.39			0.97 (0.92∼1.01)	1.02 (0.98∼1.06)
Unhealthy lifestyle	no	79.36	8.00	0.005	62.91	0.14	0.705	1	1
	yes	80.59			63.11			1.17 (1.10∼1.23)	1.27 (1.21∼1.33)
Medication adherence	regular	80.25	3.65	0.056	62.70	35.94	<0.001	1	1
	irregular	81.48			67.46			1.02 (0.94∼1.12)	0.93 (0.83∼1.01)
With CVD	no	82.22	460.11	<0.001	65.30	414.40	<0.001	1	1
	yes	71.62			52.71			1.38 (1.31∼1.46)	1.28 (1.22∼1.35)
With cancer	no	81.32	572.60	<0.001	64.03	368.73	<0.001	1	1
	yes	62.80			45.91			2.56 (2.35∼2.79)	2.06 (1.90∼2.24)
Uncontrolled BP	no	79.71	22.28	<0.001	62.57	9.42	0.002	1	1
	yes	81.40			63.91			1.01 (0.96∼1.06)	1.07 (1.03∼1.12)
Abnormal FPG	no	80.82	51.13	<0.001	63.75	72.28	<0.001	1	1
	yes	77.10			58.36			1.28 (1.20∼1.37)	1.28 (1.21∼1.35)
Dyslipidemia	no	80.67	12.30	<0.001	63.40	8.09	0.004	1	1
	yes	79.23			61.96			1.09 (1.03∼1.15)	1.05 (1.01∼1.10)
Renal dysfunction	no	80.95	337.98	<0.001	63.76	291.59	<0.001	1	1
	yes	63.60			44.10			1.94 (1.75∼2.16)	1.82 (1.64∼2.01)

Note: The adjustment factors included age, gender and urban–rural disparity in the logistic analysis.

**Figure 4.**
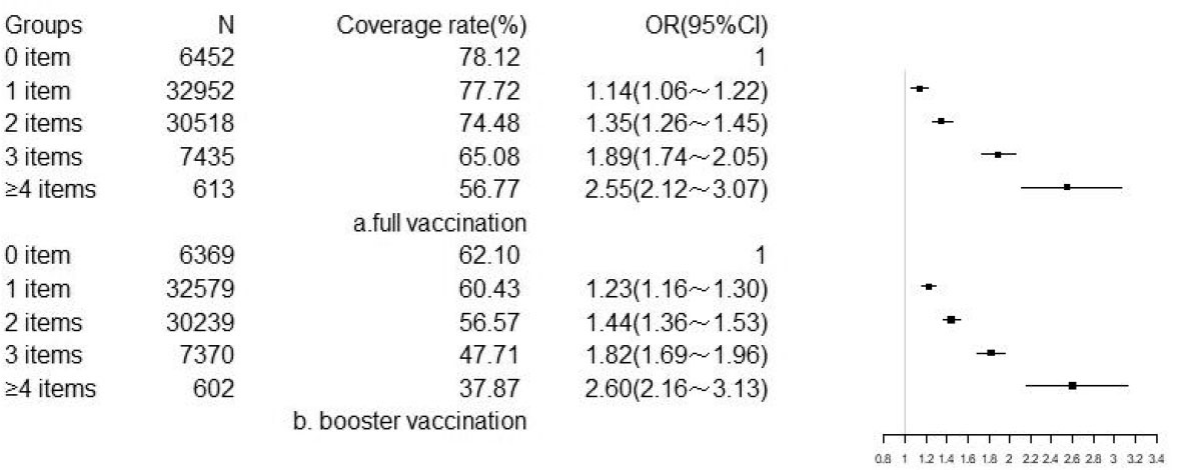
Association between number of risk factors and COVID-19 vaccination coverage among overall subjects.

**Figure 5.**
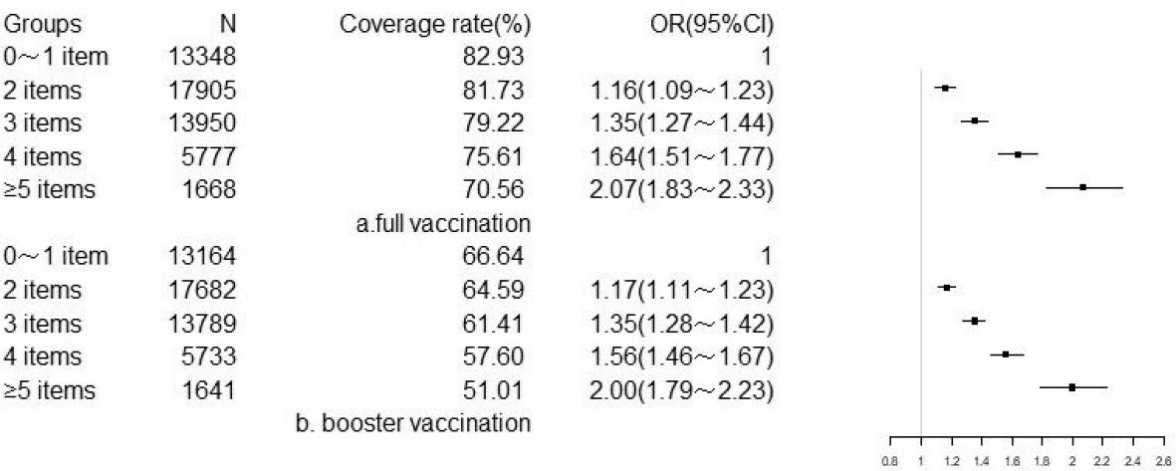
Association between number of risk factors and COVID-19 vaccination coverage in subjects who had a physical examination.

Regardless of physical examination, the coverage of COVID-19 vaccination among subjects declined with number of risk factors. For all subjects, individuals had four or more items with OR (95% CI) of 2.55 (2.12∼3.07) in full vaccination and OR (95% CI) of 2.60 (2.16∼3.13) in booster vaccination compared to those without risk factors. As the number of risk factors increased, both full and booster coverage rates demonstrated a significant decrease in proportion (wald-χ^2^=388.11, p<0.001; wald-χ^2^=382.22, p<0.001) (Figure 4). For subjects who had a physical examination, individuals had five or more items with OR (95% CI) of 2.07 (1.83∼2.33) in full vaccination and OR (95% CI) of 2.00 (1.79∼2.23) in booster vaccination compared to those with 0 or 1 risk factor. Similarly, there were significant associations between the number of risk factors and COVID-19 vaccination coverage rates (wald-χ^2^=253.86, p<0.001; wald-χ^2^=239.02, p<0.001) (Figure 5).

## Discussion

The COVID-19 vaccine coverage rates observed in this study were lower than the national average, suggesting that hypertension, as a comorbidity of COVID-19, may be associated with lower vaccination rates. A previous survey has indicated that suboptimal vaccination rates may be linked to concerns over the vaccine efficacy, apprehension about potential side effects, and fears that the vaccine itself could induce illness, particularly among individuals with chronic conditions.^17^ However, for participants who had a physical examination, vaccination rates improved significantly, with coverage reaching 80.34% for full doses and 63.07% for booster doses. Despite the government's persistent efforts to promote COVID-19 vaccination, both full and booster coverage showed growth for approximately six months after the initial launch of the vaccination campaign. The temporal trend noted in this study aligns with the data reported by the China CDC,^18^ suggesting that the initial six months is a critical period for accelerating coverage.

According to the study of enrolled hypertensive patients with a confirmed diagnosis of COVID-19 in Shanghai, China, factors such as being female, increasing age, and coexisting chronic diseases were associated with more inadequate vaccine coverage.^19^ This study showed higher coverage rates were observed in men, rural areas and younger age groups. A review reported that females were less likely to accept COVID-19 vaccination compared to males.^20^ An online cross-sectional study conducted among Chinese population showed that females with healthier lifestyles exhibited a higher COVID-19 vaccination coverage compared to males.^21^ However, the proportion of older adults was only 0.9% in that survey. A shortage of healthcare facilities, lower educational attainment, sociocultural identities and political ideologies, and a higher degree of vaccine hesitancy could result in a lower COVID-19 vaccination coverage in rural areas.^22,23^ Reasons for the inverse results in regional distribution in this study may be as follows. The Chinese government highly emphasized COVID-19 vaccination in rural areas, and conducted measures such as temporary vaccination clinics and specialized vaccination vehicles to accelerate COVID-19 vaccination. Compared with patients in urban and suburban areas, those in rural areas had the lowest rate of take-up and the least interference in terms of internet-media usage, which may be a factor because media headlines could affect awareness and interest in vaccine hesitancy and anti-vaccination during the COVID-19 pandemic.^24^

Subjects who had unhealthy lifestyles, combined with CVD and cancer showed a lower coverage in this study. Previous research has indicated that individuals with hypertension and those engaging in unhealthy behaviors such as obesity, smoking and alcohol consumption not only exhibit greater reluctance toward receiving COVID-19 vaccines but also tend to develop lower antibody levels post-vaccination.^25,26^ Concern about effectiveness and side effects are among the most prevalent reasons for COVID-19 vaccine hesitancy.^27^ In fact, there was no significant difference in the incidence of adverse events between patients with and without cardiovascular disease or cancer.^28,29^ This study also showed the inverse association between COVID-19 vaccination rates and cardiovascular risks such as uncontrolled BP, abnormal FPG, dyslipidemia and renal dysfunction in certain individuals. Although the above measurements were not a special health assessment for COVID-19 vaccination, they could reflect the health condition of patients under the EPHS program. Current technical guidelines recommend that individuals with comorbidities receive the COVID-19 vaccine when their conditions are stable and well managed.^4^ However, the reality is that high cardiovascular risk and low awareness, treatment and control rate were reported among hypertensive patients in China.^11,30^ Promoting COVID-19 vaccination in this population probably requires a comprehensive approach, including aggressive interventions for hypertension and cardiovascular risks, more accurate assessment of contraindications, and reduced concerns about the side effects of COVID-19 vaccines.

In the cardiovascular risk population, analysis of the relationship between risk factors and outcomes generally considers the cumulative effect of factors in addition to the influence of individual factors.^31^ This study used a similar approach and found that an increased number of disadvantage factors related to lower coverage of COVID-19 vaccination, regardless of whether subjects had a physical examination. A previous study had also considered the cumulative effects of 12 lifestyle behaviors, and found that a higher healthy lifestyle score could promote the COVID-19 vaccination coverage rates significantly.^21^ Despite the clear evidence that individuals with multiple risk factors are more likely to experience severe outcomes from COVID-19, there remains a paradox where these same individuals have lower vaccination rates. Data from Hong Kong, China showed that 96% of deaths occurred in people aged 60 y and older, and unvaccinated individuals had a 20 times higher risk of death compared to those who were fully vaccinated.^32^ A retrospective cohort study indicated a persistent association between hypertension and risk for severe COVID-19 illness, even after controlling for comorbidities such as chronic kidney disease, heart attack or heart failure.^33^ The causal relationship between COVID-19 vaccination and adverse events is not fully established, and severe adverse events are very rare.^34,35^ Given the current evidence, the benefits of COVID-19 vaccination significantly outweigh the risks, especially for the hypertensive population.^35^ This suggests that public health efforts should continue to focus on increasing vaccination rates among those with cardiovascular risk factors, as the potential for preventing severe illness and death is substantial.

This study had some limitations. First, subjects were hypertensive patients who were being managed by GPs in CHCs. They may have better access to medical advice, which could make them more inclined to get vaccinated against COVID-19 compared to the broader population of hypertensive patients. This could lead to an overestimation of the vaccination rate. Second, the distribution of risk factor was not equal among subjects with and without a physical examination. Despite this inequality, the study found a consistent association between single risk factors and COVID-19 vaccination coverage, suggesting that the impact of individual risk factors on vaccination rates is reliable. Third, the data in this study was collected in a partial region, and was not a national or multi-regional sample. Fortunately, the recruitment and management criteria for EPHS participants must follow the national and unified specification, and thus could ensure the representativeness and applicability of this study. In conclusion, this study initially reported COVID-19 vaccine coverage in Chinese older hypertensive patients who were managed by GPs, and showed that disparities in coverage have occurred between genders, regions and age groups, and that lifestyle, cardiovascular risk and comorbidities could influence COVID-19 vaccination coverage in hypertensive patients. These findings underscore the importance of targeted interventions to increase vaccination coverage in this vulnerable population.

## Data Availability

All the data is recorded on the electronic health platform. Authors are only authorized to develop the data but not to release it in public.
